# Evaluation of Circle of Willis variants using magnetic resonance angiography

**DOI:** 10.1038/s41598-022-21833-w

**Published:** 2022-10-20

**Authors:** Volkan Kızılgöz, Mecit Kantarcı, Şevket Kahraman

**Affiliations:** 1 Department of Radiology, Faculty of Medicine, Erzincan Binali Yıldırım University, 24100 Erzincan, Turkey; 2 Department of Radiology,, Faculty of Medicine, Atatürk University, 25240 Erzurum, Turkey

**Keywords:** Anatomy, Medical research

## Abstract

The Circle of Willis (COW) is an important collateral pathway to protect the persistence of cerebral blood perfusion. This study aims to investigate the morphological variants of this significant vascular structure with a large study population. 867 patients who had undergone MR angiography (MRA) evaluation were enrolled in this study. The MRA images of these patients obtained by the three-dimensional time-of-flight technique were re-interpreted to measure the vessel diameters of all components of the COW and classify the COW variations. In addition, correlations of the vessel calibers and the integrity of the COW with gender and age groups were presented. There was female dominance in the study population, and the mean age was 48. Type A was the most common variation in anterior (75.78%) and posterior (53.98%) circulation. Types G and H were the other common variation in the anterior circulation, and types E and D were the second and third common variations in the posterior COW, respectively. Smaller calibration for both ICAs, both P1s and BA were observed in females compared with the male group. Diameters of the BA, and both P1 segments were lower, and the left PCom diameter was significantly higher in the patients with a complete circle. There was a significant sex and age difference regarding the distribution of the complete, partially complete, and incomplete circle groups. The significant differences in the vessel calibers of specific components of the COW for complete, partial, and incomplete circulations revealed by this study should be explained with further research. In addition, meta-analyses with other studies in the literature might be a guide to understanding the morphological alterations of the COW and their relationships with a complete and non-complete circle.

## Introduction

The brain constitutes just a small percentage (2%) of the total body weight, however, it receives 20% of the total cardiac output^1^. Carotid arteries which lie anteriorly and vertebral arteries extending posteriorly in the neck, supply the blood perfusion of the brain as two major pairs of arteries. These main vessels form a vital collateral pathway among their branches. This anastomosis plays a crucial role in maintaining adequate cerebral blood flow so supply each other in case of obstruction, either in carotid or vertebral arteries. Sir Thomas Willis was the first to describe this anatomical pathway in 1664, and the name "Circle of Willis" (COW) was given to define this collateral vascular structure after him^2^. In normal conditions, there is minimal mixing of blood between the collateral branches of the circle; however, these collaterals may open up during vascular occlusive processes of the proximal feeding arteries^3^. The COW is divided into two portions, anterior and posterior portions, depending on the main arteries, which enable the brain's blood supply. Carotid arteries and their branches contribute to the anterior circulation of the COW, while the vertebrobasilar system contributes to the posterior portion^4^. Internal carotid arteries (ICAs), anterior cerebral arteries (ACAs), anterior communicating artery (ACom), posterior cerebral arteries (PCAs), basilary artery (BA), and posterior communicating arteries (PComs) form the COW. The anatomy of the COW is known to vary considerably, and functionally a complete arterial circle is a rare finding^5^. In the literature, many anatomical and radiological investigations showed normal variations of the COW in healthy individuals^4,6–10^, which were expected to occur during the process of vasculogenesis.

Magnetic resonance imaging (MRI) provides the visualization of COW using the three-dimensional (3D) images time of flight (TOF) technique, which is a non-invasive and cheaper diagnostic method compared with conventional angiography. In addition, no contrast medium is needed to obtain 3D TOF images, which can be counted as another advantage of this imaging technique.

This study aimed to reveal the frequency of each COW variation detected using TOF images of MRI in a population that had no previous cerebrovascular pathology. In addition, vessel diameters and configuration of the COW were analyzed with regard to sex and age differences. To the best of our knowledge, this study includes one of the most extensive patient series in the literature to classify the COW variants and reveal the relationships between diameters of each component and the morphological integrity of COW with sex and age differences.

## Materials and methods

Cerebral MR angiography (MRA) images of 885 patients who have undergone MR examination between January 2019 and December 2020 were re-interpreted for this observational cross-sectional study after the ethics committee approval. All of the patients in this time period who had undergone cerebral MRA for different reasons (suspicion of stroke, severe headache, dizziness, suspicion of transient ischemic attack, decreased motor strength, absent or decreased sensations, visual symptoms, memory deficit, and other positive findings of neurological examination) were re-evaluated. The institutional ethics committee confirmed our study methods to be appropriate and in accordance with relevant guidelines and regulations. Due to the retrospective nature of our study, the committee has waived consent form from each patient. All patients were examined with a 1.5-tesla MR scanner (MAGNETOM Aera, Siemens Healthcare, Erlangen, Germany). To avoid the possible effects of other vascular diseases on vessel calibers of the COW the medical records of these patients have scanned. 4 patients with left ICA occlusion, one patient with basilary artery aneurism, one patient with significant (> 50%) right ICA stenosis, one patient with ACom aneurism, one patient with vascular malformation, one patient with subdural effusion and one patient with sizeable cerebral hematoma were also excluded from the study. MR angiography images with artifacts hindering the interpretation due to motion (cannot remain stable during the examination or patients with dyskinesia) and other imaging artifacts (due to ferromagnetic intracerebral aneurism clips etc.) were excluded from the study (n = 8). After exclusions, 867 MRA images (each belonged to a different patient) with no previous history of cerebrovascular disease in our hospital records were included in the study. A 16-channel standard head coil was used in each patient. The 3D-time of flight (3D-TOF) images were handled with technical parameters as follows; TR: 24 ms, TE: 7.15 ms, flip angle: 25°, slice thickness: 0.6 mm, field of view: 243 × 256 mm, and matrix: 0.4 × 0.4 × 0.6 mm^3^. The imaging time was approximately 7 min 18 s.

**Figure 1:**
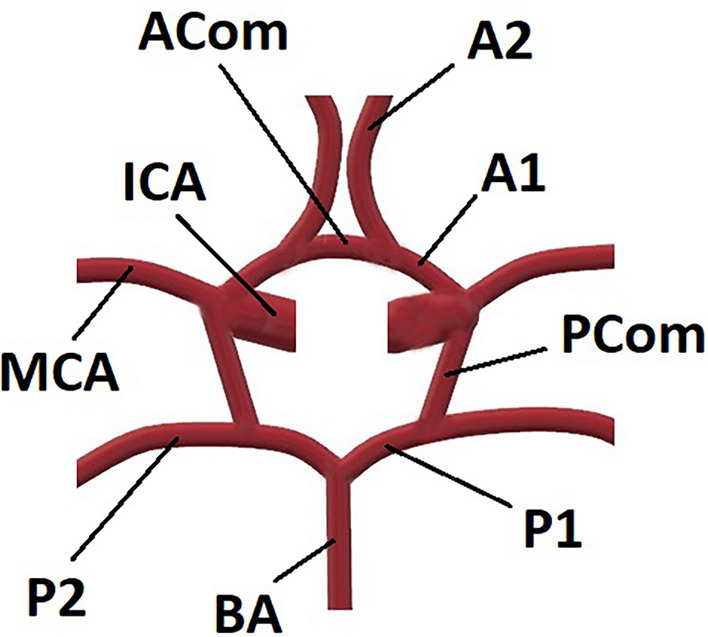
A schema will be used to present the different types of COW and to indicate the differences between the variances better (*Acom* anterior communicating artery, *PCom* posterior communicating artery, *P1* pre communicating segment of posterior cerebral artery, *P2* post communicating segment of posterior cerebral artery, *ICA* internal carotid artery, *A1* pre communicating segment of anterior cerebral artery, *A2* post communicating segment of posterior cerebral artery, *MCA* middle cerebral artery).

**Figure 2:**
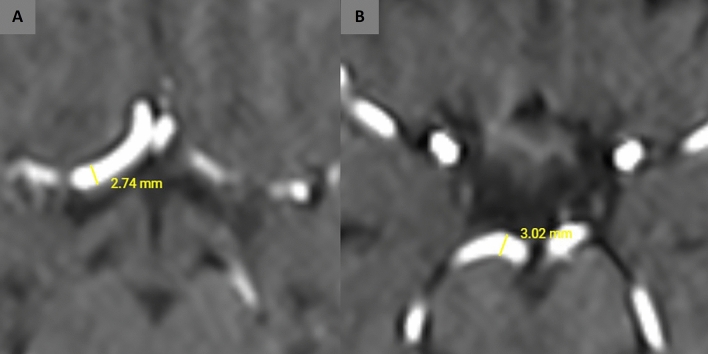
The measurements of vascular diameters of the right A1 segment (**A**) and right P1 segment (**B**) were shown. Axial MR images with ×400 magnification were used to measure the vessel calibers.

**Figure 3:**
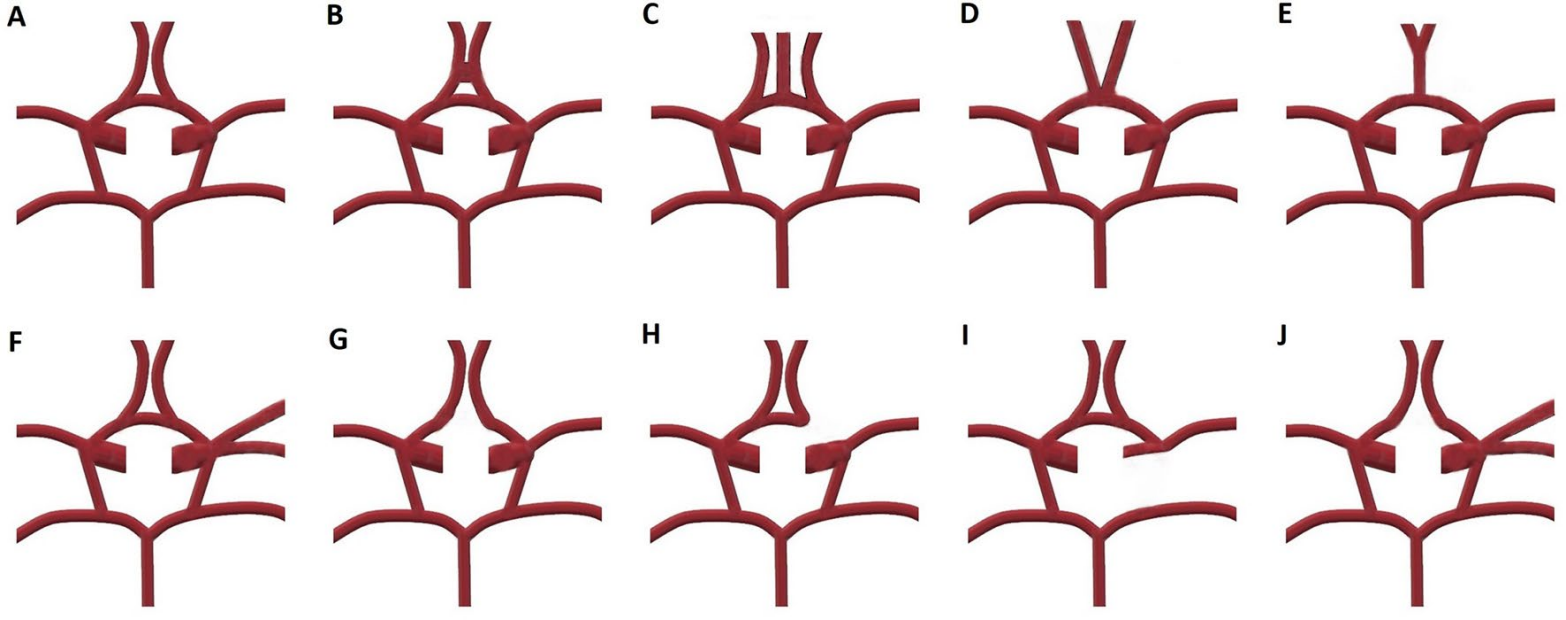
Schematic diagrams showing the morphological variations in the anterior part of the circle of Willis (COW). Type A: normal anatomical morphology in an adult. Type B: more than one AComs, Type C: the median artery of the corpus callosum arises from Acom, Type D: fusion of the anterior cerebral arteries over a short distance, Type E: A2 segments of anterior cerebral arteries are observed distally after a common trunk, Type F: the middle cerebral artery originates from the ICA as two distinct trunks, Type G: hypoplasia or absence of ACom, Type H: one of the anterior cerebral arteries have an absent or hypoplastic A1 segment, and the contralateral anterior cerebral artery gives rise to both A2 segments, Type I: hypoplasia or an absence of an ICA. The anterior cerebral artery gives rise to both A2 segments in this variance. It supplies retrograde blood flow to the ipsilateral A1 segment, Type J: hypoplasia or absence of ACom, and MCA arises as two distinct trunks (*ACom* anterior communicating artery, *ICA* internal carotid artery).

**Figure 4:**
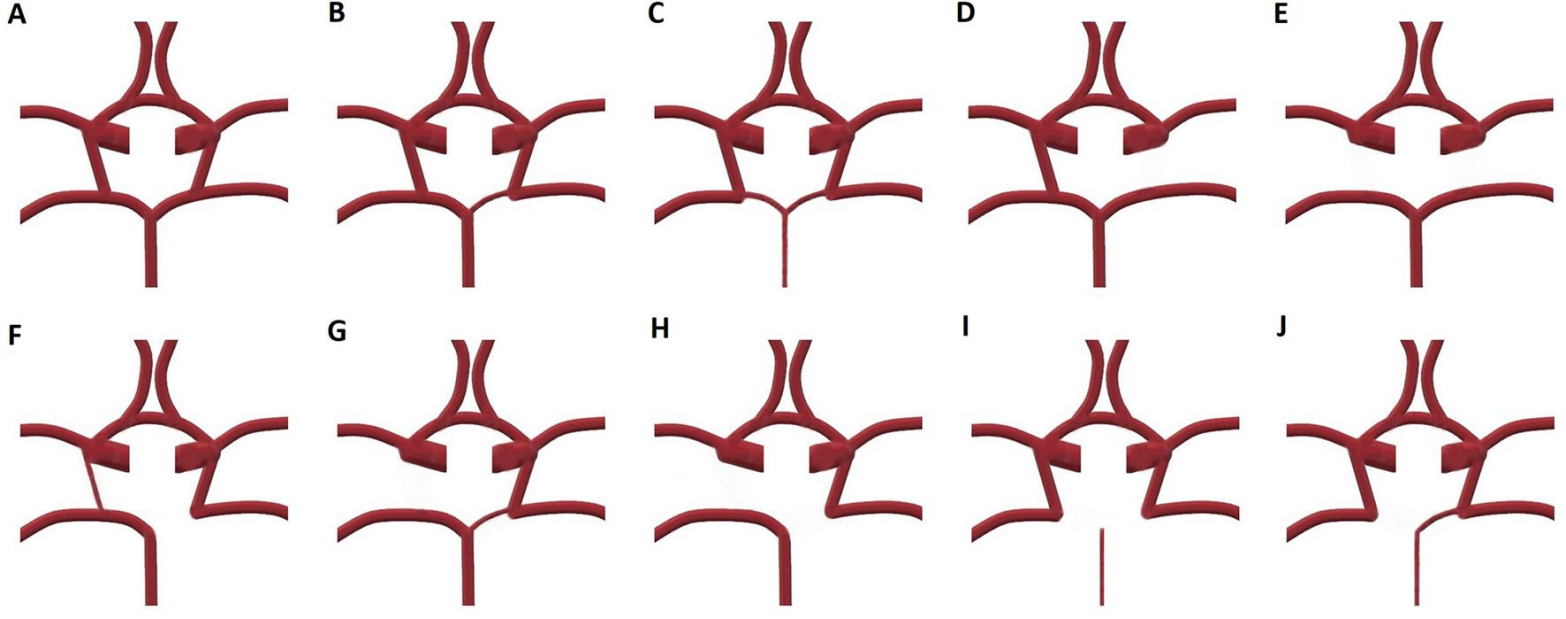
Schematic diagrams showing the anatomical variations in the posterior part of the circle of Willis (COW). Type A: normal anatomical morphology with all of the components of the COW. Type B: the posterior cerebral artery originates predominantly from the ICA. This variation has been described as unilateral fetal type PCA (where PCom has a larger diameter than P1). Type C: bilateral fetal type PCA (usually basilary artery hypoplasia accompanies this variation). Type D: absence of unilateral PCom. Type E: absence of both PComs and isolation of the posterior circulation. Type F: unilateral fetal type PCA and hypoplasia of the PCom on the contralateral side. Type G: unilateral fetal type PCA and contralateral PCom aplasia or hypoplasia. Type H: unilateral fetal type PCA and aplasia or hypoplasia of the P1 segment and PCom. Type I: bilateral fetal type PCAs with absence or hypoplasia of both P1 segments. Type J: bilateral fetal type PCAs with absence or hypoplasia of the P1 segment of either posterior cerebral artery (*PCom* posterior communicating artery, *PCA* posterior cerebral artery, *ICA* internal carotid artery).

**Figure 5:**
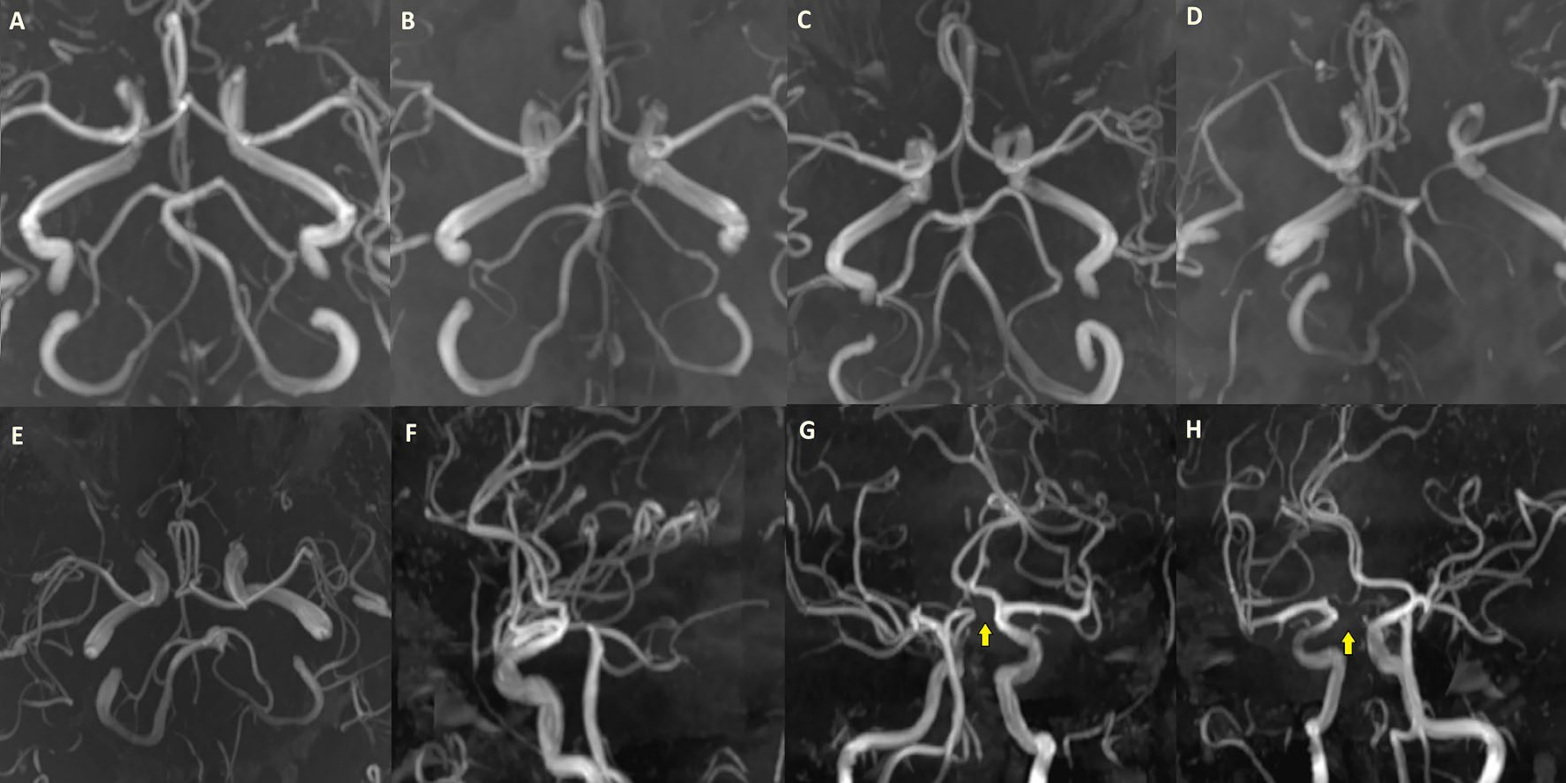
Some samples of MR angiography images from our patient population, classified as “partially incomplete” circle of Willis. Type E posterior circulation with the absence of both PComs (**A**). Type D posterior circulation with the absence of the right PCom (**B**). Type E anterior circulation with anterior cerebral arteries originating from a common trunk (**C**) Type H anterior circulation with the absence of the left A1 segment (**D**). Another patient with Type E posterior circulation (**E**) and lateral (**F**), right oblique (**G**) and left oblique (**H**) images of the same patient indicate the absence of bilateral PComs (yellow arrows).

A radiologist with 17 years of experience and a radiology resident with 4 years of experience evaluated the MRA images together concerning the presence of each vascular component of the COW, the measurement of each vessel diameters, and the classification of the COW variants using a classification system modified from Chen et al.^11^. A schematic illustration represents each variant to better understand the COW segments and how they are classified (Fig. 1). All of the measurements and evaluations were decided by consensus of both radiologists for each MRA at the same time and 400× magnification was used to prevent measurement errors and find out the exact diameter of the vessels. A picture archiving and communication system (Akgün PACS Viewer v7.5, Akgün Software, Ankara, Turkey) was used to analyze the cross-sectional images in standard digital imaging and medicine (DICOM) formats and 3D-TOF images obtained by maximum intensity projection (MIP) algorithm. A modified version of Shatri et al.’s method for measurements was used to determine the vessel calibers^12^. Vascular diameters of all segments of the COW were measured through the axial plane, 3 mm distant from the vessel origin, perpendicularly to the elongation of the artery from the inner walls. When the vascular segment is too short to use this technique, the middle part of the artery was measured (Fig. 2). The vascular segments visualized at least 0.8 mm in diameter were noted as present. As mentioned in the previous literature, the vessel segments smaller than 0.8 mm in diameter were considered hypoplastic^11,13^. The arteries without any segmental visualization or non-continuous segments were considered absent. The COW was evaluated separately as anterior and posterior compartments (Figs. 3, 4), classified, and the results were noted (Fig. 5). The integrity of the COW was classified into three groups. A complete circle (C) had no variants, and all of the components of the COW were present in these individuals. A partially complete circle (P) represented individuals with a morphological variant in either the anterior or posterior compartment. An incomplete circle (I) represented a variant in both anterior and posterior compartments of the COW. The prevalence of each anatomical variation was documented.

The patient population was also presented with age distribution, sex difference, and configuration of COW. Each anatomical classification of COW variation was shown with percentages, and the results of the vessel diameters of each component of the COW were presented on mean ± standard deviations. The vessel diameters were measured and presented according to age and sex differences. Regarding age and sex differences, independent sample t-test was carried out to calculate the significance of the difference between vessel diameters. To determine the significance of the study results between the integrity of the COW and vessel diameters, analysis of variance (ANOVA) has been used. Before analyzing the sex and age differences with regard to vessel calibers, data distribution was controlled with a Shapiro–Wilk test. There was no normal data distribution for ACom and BA, and both A1 and P1 segments to compare the sex and differences and to compare different configurations of the COW. Therefore Mann Whitney-U test was carried out to show sex and age differences. To compare the vessel diameters according to the configuration of the COW, Kruskal Wallis test was used to show any possible significant difference for these vascular segments. For each vascular segment of the COW, Spearman’s Rho test was used to analyze the correlation between age and vessel diameter. The distribution of the complete, partial and incomplete circle distributions among the populations regarding the sex and age differences was analyzed with a chi-square test. The p values < 0.05 are considered to indicate a statistically significant difference.

### Ethical approval

This study has been approved by the institutional ethics committee (Erzincan Binali Yıldırım Üniversitesi Tıp Fakültesi Etik Kurulu, Date: 07.12.2021, Session: 13, Number: E-21142744-804.01-128302) and the consent from each patient was waived by the committee due to the retrospective nature of the study.

## Results

MRA images of 867 patients were included in the study. The mean age was 48 in the study population. There was a female predominance, and the patients 20-year-old or younger constituted a relatively small group among other age groups in this study. In addition, 58.71% of the patient population had at least one variation in the anterior or posterior circulation (Table 1).

**Table 1:** Demographic and COW^*^ configuration characteristics of patients.

Patient characteristics	Number of patients (n)	Percentage (%)
**Sex**		
Female	527	60.79
Male	340	39.21
**Age in years**		
1–20	38	04.38
21–40	302	34.83
41–60	294	33.91
≥ 61	233	26.87
**Configuration of COW**		
Entirely complete (C)	358	41.29
Partially complete (P)	417	48.09
Incomplete (I)	92	10.62

*Circle of Willis.

Type A variant was dominant in the anterior circulation. Type G and type H were the other variants encountered in the patient population as second and third variations.

There was also type A predominance in the posterior circulation; however, more individuals with other variants were observed in the posterior part of the Willis (46.02%) compared with anterior circulation. Type E and type D were common variants other than type A in the posterior circulation (Table 2). On the other hand, type F and J variants in the anterior circulation and type C, H, and J variations in the posterior circulation were not observed among the study population.

**Table 2:** Prevalence of anterior and posterior COW^*^ variants.

Anterior variant type	Number of patients (n)	%	Posterior variant type	Number of patients (n)	Percentage (%)
A	657	75.78	A	468	53.98
B	10	01.15	B	2	00.23
C	9	01.04	C	0	00.00
D	2	00.23	D	140	16.15
E	17	01.96	E	239	27.57
F	0	00.00	F	10	01.15
G	143	16.49	G	2	00.23
H	25	02.88	H	0	00.00
I	3	00.35	I	1	00.12
J	0	00.00	J	0	00.00
Other	1	00.12	Other	4	00.46

*Circle of Willis.

Other variants of the COW observed during this study cannot be classified according to the classification system used in this research (Table 3). Five patients (0.58%) encountered in the current study with these unclassified variations among the whole patient population. Besides no patients observed with persistent trigeminal artery, hypoglossal artery or any other carotid-vertebrobasillar anastomoses in the study population.

**Table 3:** Other variants of the COW^*^.

Variant type	Number of patients (n)
Right PCom connecting to basillary artery	1
Absence of ipsilateral P1 segment and PCom	1
PCom aplasia with ipsilateral P1 hypoplasia	1
Right PCom without connection to P2 segment	1
Bilateral duplication of A1 segment	1

*Circle of Willis.

Smaller calibration for both ICAs, both P1s and BA were observed in females compared with the male group. The vessel diameter measurements of patients older than 40 years indicated that the diameters of ACom and left PCom were smaller, while BA and left ICA calibers were higher than the younger group (Table 4).

**Table 4:** Vessel diameters according to sex and age.

Vessel diameter (mm)	Total	Female	Male	p value	≤ 40 years	> 40 years	p value
ICA (R)	5.1 ± 0.7	4.9 ± 0.7	5.2 ± 0.7	< 0.001	5.0 ± 0.7	5.1 ± 0.7	0.193
ICA (L)	4.8 ± 0.6	4.7 ± 0.6	5.0 ± 0.6	< 0.001	4.8 ± 0.6	4.9 ± 0.6	0.027
A1 (R)	2.2 ± 0.4	2.2 ± 0.4	2.2 ± 0.4	0.553	2.3 ± 0.4	2.2 ± 0.4	0.080
A1 (L)	2.3 ± 0.4	2.2 ± 0.4	2.3 ± 0.4	0.083	2.3 ± 0.4	2.2 ± 0.4	0.057
ACom	1.5 ± 0.4	1.5 ± 0.4	1.6 ± 0.4	0.055	1.6 ± 0.4	1.5 ± 0.4	0.001
BA	3.1 ± 0.5	3.1 ± 0.5	3.2 ± 0.6	0.011	3.1 ± 0.5	3.2 ± 0.6	0.038
P1 (R)	2.1 ± 0.4	2.0 ± 0.3	2.1 ± 0.4	0.011	2.1 ± 0.4	2.1 ± 0.4	0.216
P1 (L)	2.1 ± 0.3	2.0 ± 0.4	2.1 ± 0.3	0.036	2.1 ± 0.3	2.1 ± 0.4	0.319
PCom (R)	1.6 ± 0.4	1.6 ± 0.4	1.6 ± 0.5	0.712	1.6 ± 0.4	1.6 ± 0.4	0.322
PCom (L)	1.5 ± 0.4	1.5 ± 0.4	1.5 ± 0.4	0.136	1.6 ± 0.4	1.4 ± 0.4	0.001

There was a weak and a negative correlation between vessel calibers and age for both A1, Acom and left PCom. A weak but positive correlation was observed for left ICA and BA calibration with age. There was no correlation between age and the diameters of the right ICA, both P1 segments, and right PCom segments of the COW (Table 5).

**Table 5:** Correlation between vessel diameter and age.

Vascular segment of the COW*	r and *p* values of correlation
**ICA (R)**	
r	0.051
*p*	0.133
**ICA (L)**	
r	0.087
*p*	0.011
**A1 (R)**	
r	− 0.086
*p*	0.012
**A1 (L)**	
r	− 0.094
*p*	0.006
**ACom**	
r	− 0.121
*p*	0.001
**BA**	
r	0.124
*p*	0.000
**P1 (R)**	
r	− 0.011
*p*	0.758
**P1 (L)**	
r	− 0.007
*p*	0.831
**PCom (R)**	
r	− 0.042
*p*	0.318
**PCom (L)**	
r	− 0.127
*p*	0.002

*Circle of Willis, *r* correlation coefficient.

Diameters of the BA, and both P1 segments were lower, and the left PCom diameter was significantly higher in the patients with a complete COW. In addition, lower values were obtained for ACom in the individuals with incomplete circles, and significantly lower calibers were measured for the right P1 segment in the patients with partially complete circles than the other configurations of the COW (Table 6).

**Table 6:** Vessel diameters according to configuration of COW^*^.

Vessel diameter (mm)	Complete	Partial	Incomplete	p value
ICA (R)	5.1 ± 0.7	5.1 ± 0.7	5.0 ± 0.6	0.970
ICA (L)	4.8 ± 0.6	4.9 ± 0.6	4.8 ± 0.6	0.330
A1 (R)	2.2 ± 0.4	2.2 ± 0.4	2.2 ± 0.4	0.729
A1 (L)	2.3 ± 0.4	2.3 ± 0.4	2.3 ± 0.4	0.935
ACom	1.6 ± 0.4	1.5 ± 0.4	1.2 ± 0.6	0.002
BA	3.0 ± 0.5	3.2 ± 0.5	3.3 ± 0.6	< 0.001
P1 (R)	2.0 ± 0.4	2.2 ± 0.4	2.2 ± 0.4	< 0.001
P1 (L)	2.0 ± 0.3	2.1 ± 0.4	2.2 ± 0.3	< 0.001
PCom (R)	1.6 ± 0.4	1.5 ± 0.5	1.6 ± 0.5	0.009
PCom (L)	1.5 ± 0.4	1.4 ± 0.5	1.4 ± 0.6	0.014

*Circle of Willis.

There was a significant sex and age difference regarding the distribution of the complete, partially complete, and incomplete circle groups. A higher percentage of complete circles were observed in females and in the group younger than 40 years old (Table 7).

**Table 7:** Sex and gender distribution according to configuration of the COW^*^.

Sex and age variables	Number of patients	Configuration of COW*			p value
		Complete	Partial	Incomplete	
**Sex**					
Female	527	238 (45.16%)	233 (44.21%)	56 (10.63%)	0.011
Male	340	120 (35.29%)	184 (54.12%)	36 (10.59%)	
**Age**					
≤ 40 years	340	184 (54.12%)	140 (41.18%)	16 (04.71%)	< 0.001
> 40 years	527	174 (33.02%)	277 (52.56%)	76 (14.42%)	

*Circle of Willis.

## Discussion

The morphological configuration of the COW has been studied by many anatomical and clinical studies in the past^14–16^. Studies on larger populations undoubtedly will have attention in the literature since the larger the sample size; the more accurate and valuable information is obtained. As one of the most extensive series in the literature, this study aimed to be descriptive and informative about the morphological variances of the COW.

There are six pairs of branchial arch arteries playing a role in the formation of cerebral circulation. Early in the embryologic period, internal carotid arteries (ICAs) are formed by the contribution of third branchial arch arteries. Ventral pharyngeal arteries fuse with the ICAs to form the common carotid arteries (CCAs). ICA branches into anterior and posterior divisions around 28 days of development. Anterior cerebral artery (ACA), middle cerebral artery (MCA), and anterior choroidal artery are formed by anterior ICA. Carotid-vertebrobasilar connections (trigeminal artery, otic artery, hypoglossal artery and the proatlantal artery) provide the vascular supply of the hindbrain. Beginning with the formation of posterior communicant arteries (PComs) which connects to distal portion of the basillary artery (BA), the trigeminal artery, otic artery, and hypoglossal artery regress but the proatlantal artery remains until the vertebral arteries develop. Middle cerebral artery begins to develop around 35 days from the anterior division of the ICA. In this time period, ACA grows medially and anterior communican artery (ACom) develops. The ACA and ACOM is observed in COW, typically at 6–7 weeks of development^17^.

All components of a complete COW have been considered to have the following vessels; an ACom, two A1 segments, two PComs, two P1 segments, two ICAs, and one BA, as studied in earlier investigations in the literature. Among all other anatomical variations of the anterior circulation, there was a type A dominance in most of the studies in the past, similar to the results of this new investigation. The prevalence of the complete anterior circulation ranged between 74 and 90%, while this prevalence was lower in the autopsy series^4^. 75.78% of the study population in this current research was found to have a complete circle, similar to other studies in the literature.

There are three main categories of the posterior circulation mentioned in the literature, and posterior circulation was named as adult type, transitional type, and fetal type according to this classification. In the adult type, the diameter of the P1 segment is larger than the PCom diameter. In the transitional type, diameters of both P1 and PCom are equal (and equally contributed to the P2 segment formation of the PCA. In the fetal or embryonic type of the posterior configuration, the diameter of the P1 segment is smaller than the diameter of the PCom and P2^16^. This situation played an important role in determining the posterior circle variants. In some previous studies, type E predominance (in which both PComs were absent) was observed in the posterior circulation^3,18,19^. The results of the current study showed similarities with some other studies in the past regarding the posterior circulation, and there was type A predominance in the study population, while type E was the second common variance^1,20^.

In the literature, there are studies in which some of the variants were not observed. In Keeranghat et al.’s study F and I types in the anterior circulation were not encountered^1^. In Maaly et al.’s study there was no patients with I type in the posterior circulation^18^. I variant in anterior and posterior, J type in anterior circulation has not been observed in another study^4^. No patients with I type in anterior and posterior, or J and F type in anterior circulation has been reported in the study population of another research^3^. In Shaikh et al.’s research, F, I and J types in the anterior circulation were not encountered^19^. In this current study, there was no patient with F or J variants of the anterior circulation, in addition, no C, H or J variations were observed in the posterior circulation among the patient population.

The percentage of patients with a partially complete circle was the highest, and patients with an incomplete circle were the lowest in this current study, similar to Keeranghat et al.'s research. In some other previous studies, the entirely complete circle was the most common configuration of the COW^3,18^. Unlike Keeranghat et al.'s study, no difference was observed between age groups regarding the distribution of these configuration types; however, there was a significant difference between females and males in the current study. Maaly et al.'s study indicated a higher percentage of entirely complete circulation in males and the age group older than 40 years, unlike this current research.

The diameters of both ICAs, both P1 and BA segments were higher, in males than females in this research. In another study, higher left PCom and left ICA diameters were measured in males^1^. The ICA, BA, A1, and Acom diameters were significantly higher in males in another research in the literature^18^. Moreover, ICA, BA, A1, and PCom diameters have been found significantly higher in the age group younger than 40 years in the same study. In the study population older than 40 years, the diameter measurements of the right P1 had lower, and the right PCom had higher values in another previous study^1^. In the study population of the current research, the diameters of ACom and left PCom were significantly smaller, while BA and left ICA calibration were higher in patients older than 40 years compared with younger individuals. Older age can associate with an increase in the thickness of the vessel wall and therefore a reduction in the lumen. Therefore a negative correlation is expected between age and vessel caliber. However, correlation analysis indicated this relationship only for both A1s, Acom, and left PCom segments of the COW. In this research, the diameters of the BA and both P1 segments were significantly lower and the left PCom diameter was higher for the individuals with an entirely complete circle. The calibration of the ACom was significantly lower for the individuals with incomplete circles compared with complete and partially complete circles. P1 segment was also measured with a lower caliber in the partially complete COW group. There was a significant sex and age difference regarding the distribution of the configuration of the COW. A higher percentage of complete circles were observed in females than in males. In addition, the individuals who were 40 years old and younger were observed with a higher percentage of complete circles than the older group. In a previous study, the diameters of the right P1, both PComs, right ICA, and both A1 segments were found to be significantly different concerning the configuration of the COW. Similar to our study, there was a significant sex and age difference regarding the distribution of the different configurations of the COW, however, the partially complete circle group was observed in a higher percentage in all groups in the study^1^.

There were limitations of this study to be mentioned since the data and the descriptive analysis in this study should be interpreted carefully with the awareness of these limiting factors. First, the 3D TOF sequence is not a perfect imaging technique for analyzing and measuring vascular calibrations. Although this technique is widely used and very successful in imaging intracerebral circulation, this technique has some difficulties in imaging small vascular collateral channels because of turbulent flow or slower velocity of blood adjacent to the vessel wall due to the laminar flow^21^. All of the components of the COW were measured by 400× magnification to prevent measurement errors and find the exact diameter of the vessels; however, even with a careful and elaborate technique, the human error factor should also be considered a limitation of this study. In some of the studies in the literature, 1 mm vessel caliber is considered to determine the hypoplasia of the vascular segments^1,20^, while some others have used 0.8 mm^4,18^ as in this current research. Considering this situation, a comparison of the COW variance studies should be made carefully. Some researchers indicated indifferent results from similar topics studied on this subject in the literature. This situation might be related to genetic differences among different populations. It might be helpful to classify the results of these studies concerning specific demographic features of each investigation, which might be another topic of a review article. The researchers avoided expressing the possible effects of other vascular conditions on statistical results and included the patients without any other cerebrovascular lesions or positive history of cerebrovascular diseases. Especially, ICA status has a known effect on the vessel calibers of the COW^22,23^. The measurements would reflect more precise results if all the patients had a known ICA status. Some of the statistical calculations were carried out with non-parametric tests due to the data distribution properties. For this reason, the results of the study should be evaluated carefully. Moreover, even with this study's considerable sample size, more patients should be re-interpreted retrospectively to analyze and calculate more accurate values to better understand the incidences of these variations.

## Conclusion

A lower percentage of the complete circle was observed in males, and the statistically significant difference between female and male populations regarding the distribution of complete, partially complete, and incomplete circulations may result from the male population having more variations. For the whole study population, the differences between vessel diameters of complete, partially complete, and incomplete circles might reflect altered flow dynamics in the COW. The specific vessel caliber differences indicated in this study may be compared with other studies in the literature and meta-analyses with larger study populations might be a guide to better understanding the morphological alterations in the COW and studying the differences between complete and non-complete circles.

## Data Availability

The data that support the findings of this study are available from the corresponding author upon reasonable request.
